# Immediate Neoumbilicoplasty Following Excision of a Symptomatic Umbilical Lipoma Using Local Tissue Rearrangement and Fascial Fixation: A Case Report

**DOI:** 10.7759/cureus.115257

**Published:** 2026-08-26

**Authors:** Ludmila Rodrigues Ribeiro da Silva

**Affiliations:** 1 Burn Reconstructive Surgery, Shriners Hospitals for Children, Galveston, USA

**Keywords:** benign lipoma, lipoma, lipoma excision, neoumbilicoplasty, rare site of lipoma, umbilical lesion, umbilical problems

## Abstract

Umbilical tumors are uncommon and may present with pain, progressive enlargement, cosmetic deformity, and diagnostic uncertainty. Although lipomas are among the most common benign soft-tissue tumors, symptomatic lipomas involving the umbilical region and requiring excision with immediate reconstruction are rarely reported.

A 43-year-old woman presented with a progressively enlarging, painful, nodular, and hyperpigmented umbilical lesion associated with distortion of the native umbilical anatomy. The clinical appearance raised concern for a melanocytic malignancy, including melanoma, prompting complete en bloc excision for definitive histopathological evaluation followed by immediate neoumbilicoplasty using a standardized geometric preoperative design, local tissue rearrangement, and deep fascial fixation to recreate umbilical depth and contour.

According to the official pathology report, histopathological examination confirmed a benign lipoma without evidence of atypia or malignancy. Postoperative recovery was uneventful, without infection, necrosis, wound dehiscence, or recurrence. An umbilical conformer was used postoperatively in conjunction with abdominal compression to provide external support during scar maturation. At six-month follow-up, the neoumbilicus demonstrated preserved depth and contour, satisfactory scar maturation, and a favorable aesthetic result, with the patient reporting high satisfaction with the reconstructed umbilicus. This case illustrates that immediate neoumbilicoplasty using local tissue rearrangement and deep fascial fixation may represent a practical reconstructive option following excision of selected benign umbilical lesions and highlights the value of structured surgical planning when excision compromises the native umbilical anatomy.

## Introduction

The umbilicus is a defining aesthetic landmark of the anterior abdominal wall and contributes substantially to abdominal symmetry, body image, and overall aesthetic appearance. Although it has little physiological function after birth, loss, distortion, or absence of the umbilicus may result in significant cosmetic dissatisfaction and psychological distress despite otherwise successful abdominal wall reconstruction. Consequently, preservation or restoration of a natural-appearing umbilicus has become an important goal of reconstructive surgery. The ideal neoumbilicus should demonstrate adequate depth, superior hooding, vertical orientation, minimal visible scarring, and harmonious integration with the surrounding abdominal contour while maintaining long-term stability over time [1-6].

Umbilical reconstruction has evolved considerably over the past several decades, with numerous techniques described for patients undergoing abdominoplasty, ventral hernia repair, oncologic resection, trauma reconstruction, congenital abdominal wall reconstruction, and secondary revision procedures [1-5]. Although a wide variety of reconstructive methods have been proposed, including purse-string techniques, local advancement flaps, V-Y flaps, C-V flaps, island flaps, and combinations of local tissue rearrangement with fascial fixation, no single technique has been universally accepted as the gold standard [2-5]. Instead, successful reconstruction depends on restoration of a natural umbilical shape and depth while preserving vascularity, minimizing visible scars, and achieving durable fixation to the abdominal fascia [2-6].

Primary umbilical tumors are uncommon, and the differential diagnosis of an umbilical mass encompasses congenital anomalies, umbilical hernias, epidermoid cysts, endometriosis, inflammatory lesions, metastatic tumors such as Sister Mary Joseph nodules, and benign mesenchymal tumors including lipomas [7-10]. Although lipomas are among the most common benign soft-tissue tumors in adults, involvement of the umbilical region and central abdominal midline is infrequently described in the literature. Published reports addressing lipomas specifically arising within or distorting the umbilical unit are limited, and the true incidence of this presentation has not been well established. This uncommon location is clinically relevant because a lipomatous lesion presenting as an umbilical mass may enter the differential diagnosis with more frequently encountered conditions, particularly an umbilical hernia. Careful clinical examination is therefore important in determining whether the lesion appears confined to the superficial soft tissues or is associated with an underlying abdominal wall defect. Progressive enlargement, pain, cosmetic deformity, diagnostic uncertainty, or concern for malignancy may justify complete surgical excision [7-10].

When complete excision results in partial or complete loss of the native umbilicus, immediate reconstruction may offer the advantage of restoring abdominal aesthetics during the same operation while avoiding a secondary reconstructive procedure [2-6,11,12]. Nevertheless, reports describing immediate neoumbilicoplasty following excision of symptomatic benign umbilical tumors remain scarce, with most published studies focusing on reconstruction performed during cosmetic abdominoplasty, abdominal wall reconstruction, or oncologic surgery rather than benign umbilical masses [2-6,11,12].

This report describes the successful management of a progressively enlarging symptomatic umbilical lipoma treated by complete surgical excision followed by immediate neoumbilicoplasty using local flap rearrangement and fascial fixation. In addition to presenting the clinical outcome, this report provides a detailed description of the operative technique, including the surgical design and measurements used to recreate a natural-appearing umbilicus, and discusses the rationale for immediate reconstruction in light of the current literature.

## Case presentation

A 43-year-old woman presented with a progressively enlarging and painful umbilical lesion associated with localized discomfort for several months. She reported progressive distortion of the native umbilical anatomy and intermittent tenderness, particularly during daily activities and local pressure.

Physical examination demonstrated a well-circumscribed, superficial, nodular, and hyperpigmented lesion involving the umbilical region, closely associated with the overlying skin and producing distortion of the native umbilicus. The nodular morphology and prominent pigmentation, together with progressive enlargement, raised clinical concern for a melanocytic malignancy, including melanoma. There was no clinical evidence of an associated umbilical hernia or abdominal wall defect. Given the superficial location of the lesion and the absence of clinical findings suggestive of an underlying hernia, preoperative ultrasonography was not obtained. Because of progressive enlargement, persistent pain, cosmetic deformity, and concern for malignancy, complete surgical excision was indicated to obtain a definitive histopathological diagnosis.

**Figure 1:**
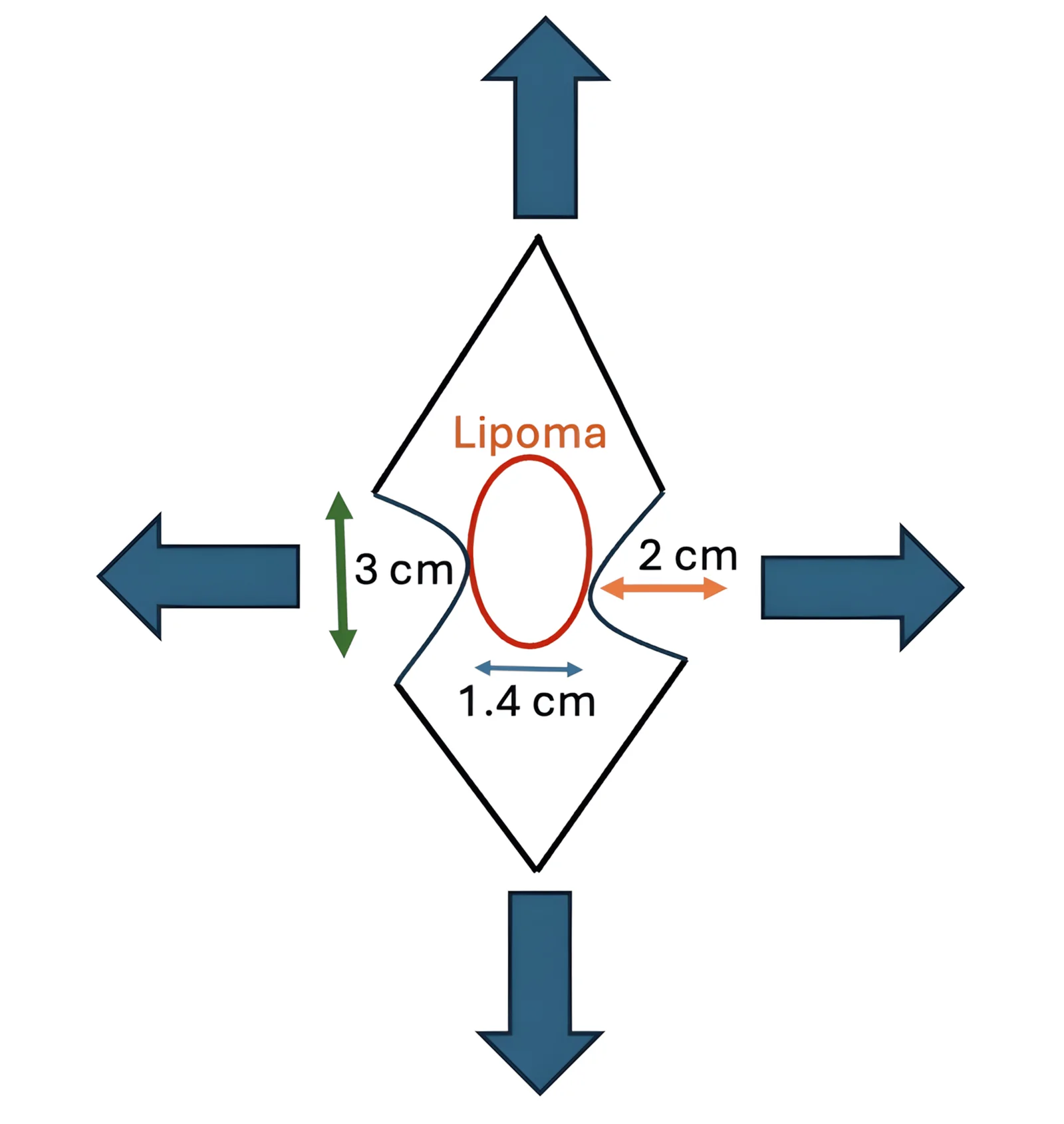
Schematic illustration of the preoperative surgical design. The schematic demonstrates the symmetric biconcave skin design centered on the umbilical lipoma and the planned local tissue rearrangement. The design measured approximately 3 cm along the vertical axis and 2 cm at the maximum horizontal width of each lateral curvilinear flap. The central lesion measured approximately 1.4 cm in transverse dimension. The large blue arrows indicate the vertical and horizontal reference axes used to guide geometric alignment and symmetry during preoperative planning. This standardized design was subsequently transferred to the patient for immediate neoumbilicoplasty following lesion excision.

**Figure 2:**
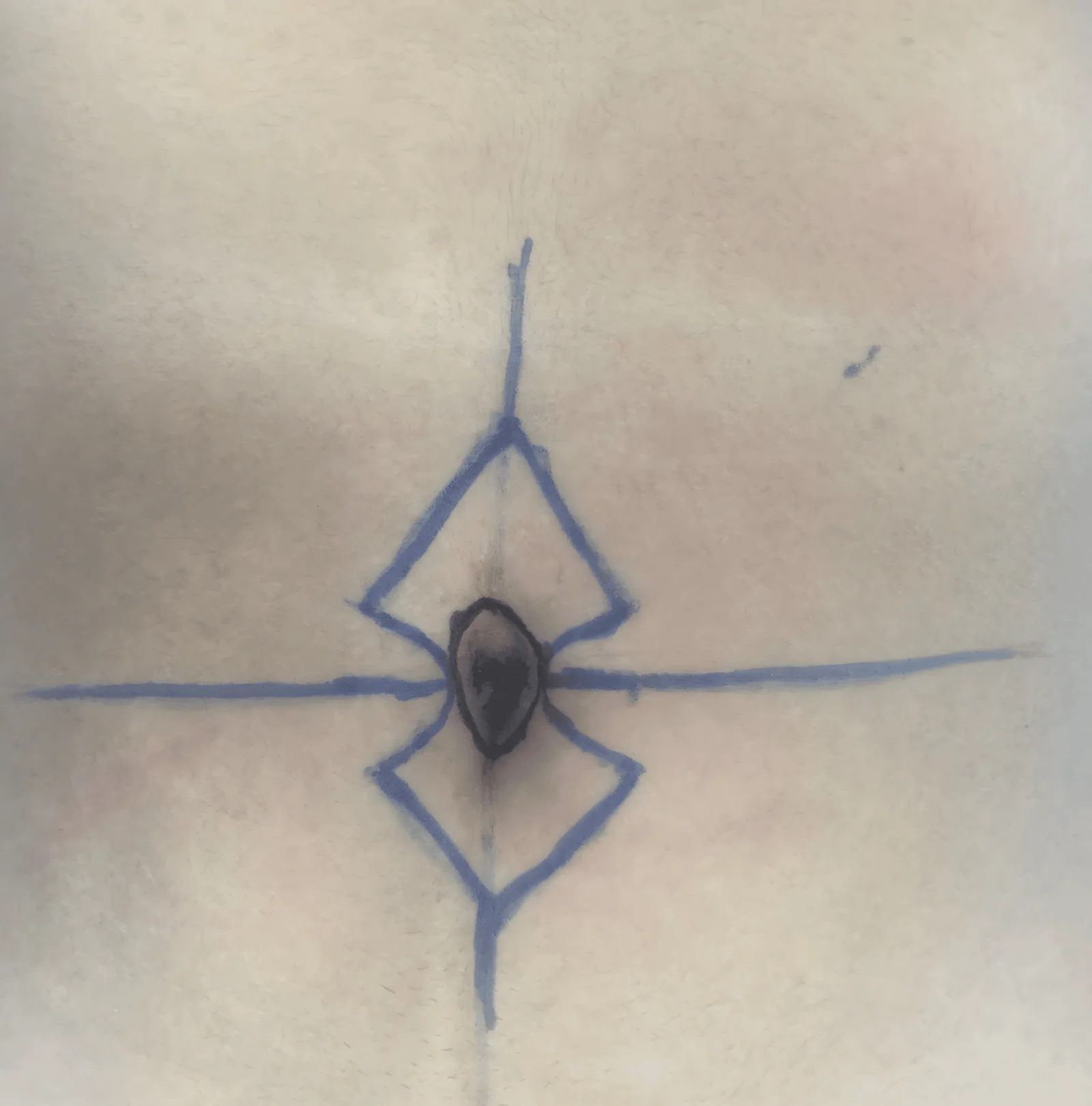
Preoperative surgical markings for immediate umbilical reconstruction. Preoperative view demonstrating the superficial nodular and hyperpigmented umbilical lesion and the geometric surgical markings designed for en bloc excision and immediate neoumbilicoplasty. The clinical appearance of the lesion, including its nodular morphology and prominent pigmentation, contributed to preoperative concern for a melanocytic malignancy.

A schematic preoperative design was created to illustrate the planned skin excision and local tissue rearrangement for immediate neoumbilicoplasty (Figure 1). The design was constructed along defined vertical and horizontal reference axes to maintain symmetry and was subsequently transferred to the patient during preoperative marking (Figure 2). A symmetric biconcave design with bilateral curvilinear flaps was used, measuring approximately 3 cm along the vertical axis and 2 cm at the maximum horizontal width of each lateral curvilinear flap.

Given the preoperative concern for malignancy, the lesion and involved overlying skin were excised en bloc with a surrounding margin of grossly uninvolved tissue to avoid leaving residual lesional tissue and to permit complete histopathological evaluation. Following excision, the bilateral curvilinear flaps were brought toward the midline to assess the amount and distribution of residual skin redundancy before definitive closure. The tissues were temporarily approximated, and areas of excess skin were reassessed and remarked intraoperatively. Additional redundant skin was then excised as required to permit smooth redraping of the surrounding abdominal skin and minimize residual contour irregularities and dog-ear formation.

The preserved local flaps were subsequently rearranged to reconstruct the umbilical unit. To recreate and maintain the central umbilical depression, the reconstructed umbilicus was anchored to the underlying abdominal fascia using three simple interrupted 3-0 nylon sutures. The surrounding tissues were then redraped and approximated, with final adjustment of the skin edges based on the intraoperative assessment of tissue redundancy. This sequential approach allowed the extent of skin excision to be determined after lesion removal and flap approximation rather than solely from the dimensions of the original lesion. Final closure was performed after confirming satisfactory abdominal contour, symmetry, and neoumbilical depth.

According to the official pathology report, gross examination demonstrated an ellipse of skin and subcutaneous tissue measuring 6.5 × 4.3 × 3.0 cm, with a 1.4 × 0.4 cm cutaneous depression. On sectioning, the specimen showed alternating whitish-brown, firm-elastic tissue and yellowish, soft tissue. Histopathological examination subsequently confirmed a benign lipoma composed of mature adipose tissue without atypia or malignancy.

**Figure 3:**
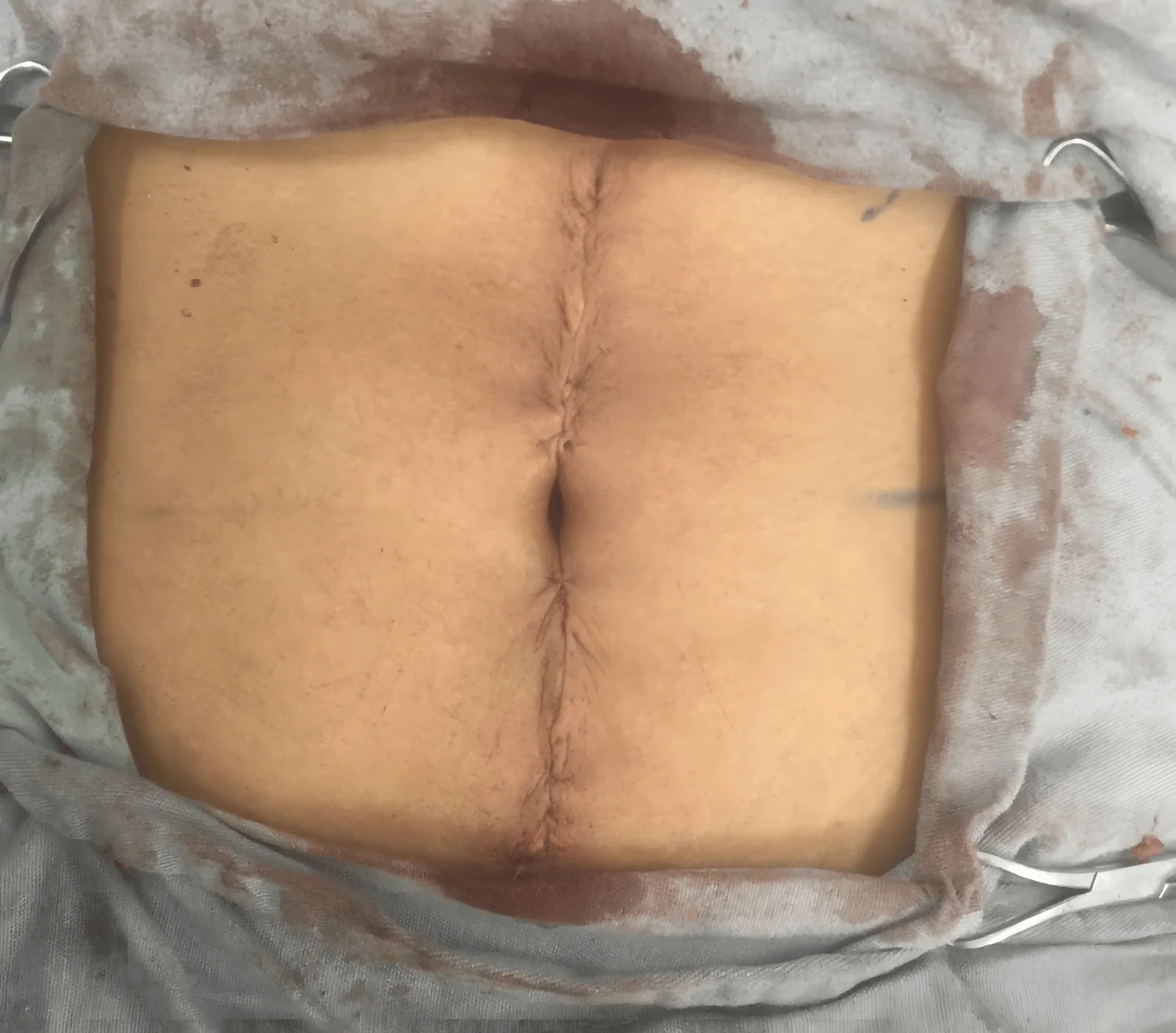
Immediate postoperative appearance following umbilical reconstruction. Immediate postoperative view following en bloc excision of the symptomatic umbilical lesion and single-stage neoumbilicoplasty, demonstrating restoration of umbilical contour and abdominal aesthetics.

**Figure 4:**
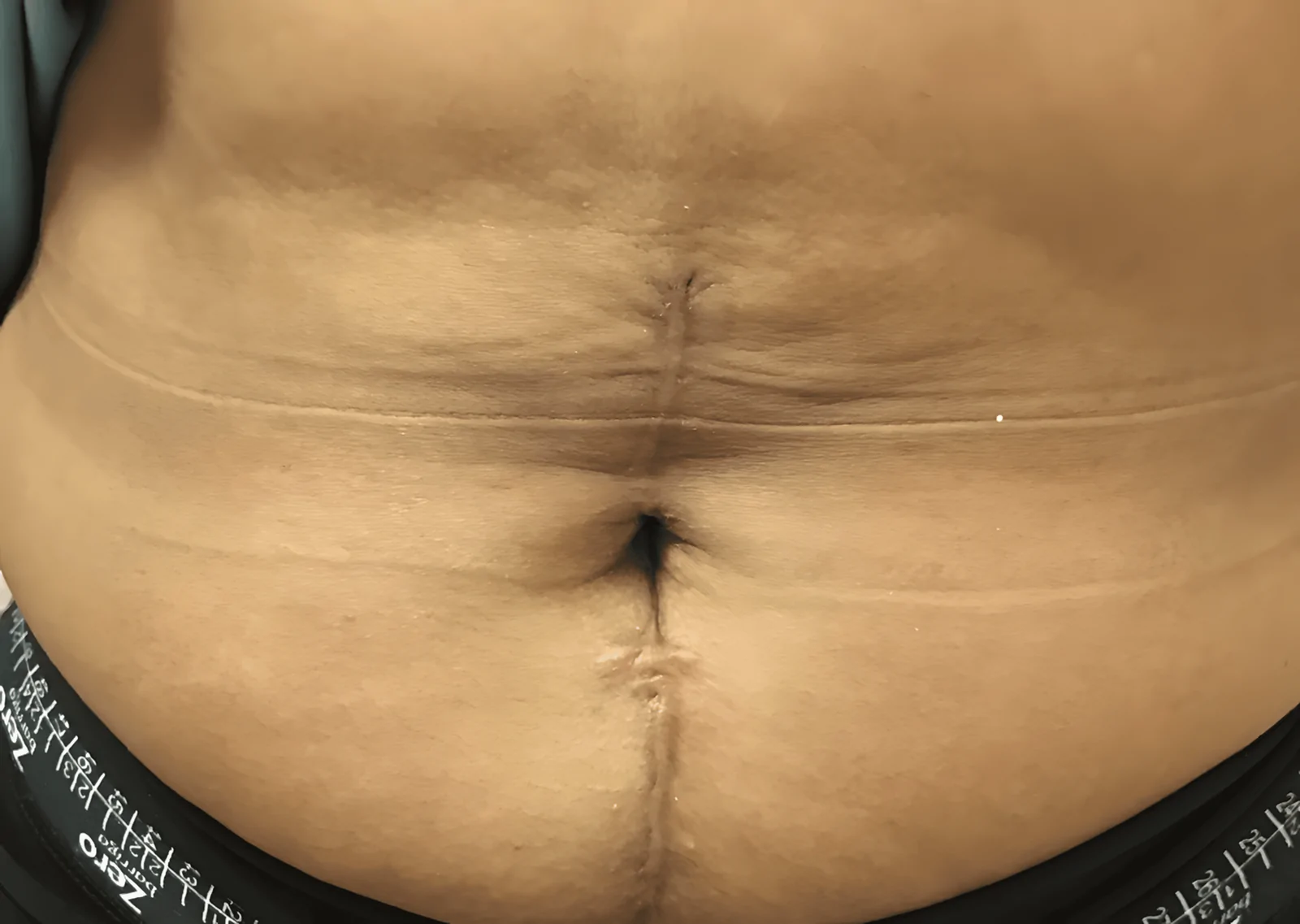
Six-month postoperative follow-up after neoumbilicoplasty. Six-month postoperative follow-up demonstrating a well-healed neoumbilicoplasty with satisfactory scar maturation, preserved neoumbilical depth and contour, and favorable integration with the surrounding abdominal wall. During the postoperative period, an umbilical conformer was used in conjunction with a compression abdominal binder to provide external support and assist in maintaining the shape and depth of the reconstructed neoumbilicus during scar maturation.

The immediate postoperative appearance demonstrated successful reconstruction of the neoumbilicus with satisfactory contour and preservation of umbilical depth (Figure 3). The postoperative course was uneventful, with no evidence of wound infection, skin necrosis, wound dehiscence, or recurrence. An umbilical conformer was used postoperatively in conjunction with a compression abdominal binder to provide external support and assist in maintaining the shape and depth of the reconstructed neoumbilicus during scar maturation. The six-month postoperative result is shown in Figure 4. 

At six-month follow-up, the reconstructed umbilicus maintained satisfactory depth, vertical orientation, and contour, with no evidence of stenosis, skin necrosis, infection, wound dehiscence, or recurrent mass. The patient reported being highly satisfied with the aesthetic outcome and specifically stated that she preferred the appearance of the reconstructed umbilicus to that of her native umbilicus before development of the lesion.

## Discussion

The umbilicus is a relatively small anatomical structure but represents a major aesthetic landmark of the anterior abdominal wall. Its position, depth, orientation, and surrounding contour contribute substantially to the perception of abdominal symmetry and overall aesthetic appearance. Consequently, loss or distortion of the native umbilicus may remain conspicuous even after an otherwise successful abdominal operation [1-6]. These considerations are particularly relevant when complete excision of an umbilical lesion requires partial or total sacrifice of the native umbilical architecture.

Numerous techniques for neoumbilicoplasty have been described, reflecting the absence of a universally accepted reconstructive method [1-5]. Available approaches include purse-string techniques, single- and multiple-flap reconstructions, local tissue rearrangement, skin grafting, and scarless or minimally scarred techniques. A recent systematic review of neoumbilical reconstruction demonstrated generally favorable aesthetic outcomes and relatively low complication rates across several approaches while emphasizing that technique selection should be individualized according to the operative setting, available tissue, and reconstructive requirements [2]. An aesthetically favorable neoumbilicus should reproduce the three-dimensional characteristics of the native umbilicus rather than merely create a cutaneous opening, with adequate depth, a predominantly vertical configuration, subtle superior hooding, minimal visible scarring, and harmonious integration with the surrounding abdominal contour [1,3,6].

The technique used in the present case was designed around these principles. A symmetric biconcave preoperative design centered on the lesion permitted complete excision while preserving sufficient adjacent skin for immediate reconstruction. Defined vertical and horizontal reference axes and standardized measurements were used to maintain geometric symmetry. Following lesion excision, the bilateral flaps were temporarily approximated to assess residual skin redundancy, allowing the skin to be reassessed and remarked before definitive excision of excess tissue. Local tissue rearrangement was then combined with deep fixation of the reconstructed umbilicus to the abdominal fascia using three simple interrupted 3-0 nylon sutures, creating a stable central depression without requiring a distant donor site. This sequential reassessment allowed the final skin excision to be adapted to the post-excisional defect and surrounding tissue redundancy rather than being determined solely by the dimensions of the original lesion.

The importance of deep fixation and controlled three-dimensional contour has been emphasized in several previously described techniques. Yousif et al. incorporated dermal fixation to the underlying fascia as a central component of dermal-shift reconstruction [13], while Silva Júnior and de Sousa used multiple fixation sutures and manipulation of surrounding adipose tissue to enhance umbilical depth in a series of 108 patients [14]. Ngaage et al. similarly used deep tacking sutures between the dermis and abdominal fascia in a two-step technique and reported that inadequate indentation was the principal concern among patients who were less satisfied with their results [15]. More recently, Netto et al. described fixation between the abdominal wall dermis and rectus sheath to create a neoumbilical depression [16], and Cala Uribe et al. emphasized precise preoperative marking and structured surgical execution in their six-step “house-roof” technique [17]. Collectively, these reports support the importance of geometric planning, controlled tissue rearrangement, and stable deep fixation in creating and maintaining neoumbilical depth and contour [13-17].

The standardized preoperative schematic presented in this report provides an additional educational component of the technique. Rather than relying solely on intraoperative visual estimation, the reconstruction was planned along defined vertical and horizontal reference axes, with approximately 3 cm along the vertical dimension and a maximum horizontal width of 2 cm for each curvilinear flap. These measurements are not proposed as universal dimensions for neoumbilicoplasty but instead illustrate the geometric principles applied in this patient. Flap dimensions should be individualized according to lesion size, abdominal anatomy, skin laxity, and the amount of local tissue available for reconstruction.

Immediate reconstruction was selected because complete removal of the lesion would substantially alter the native umbilical anatomy. Performing neoumbilicoplasty during the same operation allowed definitive treatment of the lesion and restoration of abdominal contour without requiring a secondary reconstructive procedure. It also permitted incorporation of the surrounding local tissues into the reconstruction before secondary scar contraction occurred. Published reconstructive series demonstrate that immediate neoumbilicoplasty can be incorporated into a variety of abdominal procedures when adequate local tissue is available and careful planning is performed [2,13-17].

Although laparoscopic approaches may be appropriate for umbilical pathology associated with an abdominal wall defect, hernia, or intra-abdominal disease, the lesion in the present case was clinically superficial and closely associated with the overlying skin, with no clinical evidence of an associated umbilical hernia or abdominal wall defect. Complete excision required removal of the involved superficial tissues, resulting in a defect that required reconstruction of the native umbilical unit. An open approach therefore provided direct access for en bloc excision while simultaneously allowing assessment of residual skin redundancy, local tissue rearrangement, deep fascial fixation, and restoration of the umbilical contour. In this setting, a laparoscopic approach would not have addressed the superficial soft-tissue component or avoided the need for external reconstruction and therefore offered no clear reconstructive advantage.

Although lipomas are common benign soft-tissue tumors, involvement of the umbilical region is infrequently encountered and may create both diagnostic and reconstructive challenges. The differential diagnosis of an umbilical mass is broad and includes hernias, epidermoid lesions, endometriosis, inflammatory processes, congenital remnants, and metastatic lesions [7-10]. In the present case, the superficial lesion demonstrated a nodular and hyperpigmented appearance in addition to progressive enlargement, pain, and distortion of the native umbilical anatomy. These clinical characteristics raised concern for a melanocytic malignancy, including melanoma, and supported complete en bloc excision with a surrounding margin of grossly uninvolved tissue to permit definitive histopathological evaluation. Histopathological examination subsequently confirmed a benign lipoma composed of mature adipose tissue without atypia or malignancy. Published literature specifically addressing immediate neoumbilicoplasty after excision of benign umbilical tumors remains limited, as most reports of neoumbilical reconstruction involve abdominoplasty, hernia surgery, abdominal wall reconstruction, breast reconstruction donor sites, congenital conditions, or secondary reconstruction [1-5,13-17]. The present case therefore represents a less frequently reported indication in which complete excision of a benign umbilical lesion and aesthetic reconstruction of the umbilical unit were performed during the same operation.

Maintenance of neoumbilical depth during scar maturation represents another important consideration. Several authors have described temporary postoperative support, including petrolatum gauze packing, Xeroform bolsters, or tie-over dressings, to support the newly created umbilical depression during early healing [13,15-17]. In the present case, a removable umbilical conformer was used postoperatively in conjunction with an abdominal compression binder to provide external support and assist in maintaining neoumbilical depth and contour during scar maturation. At six-month follow-up, depth and contour remained preserved without stenosis, skin necrosis, infection, wound dehiscence, or recurrent mass. The patient also reported being highly satisfied with the aesthetic result and stated that she preferred the appearance of the reconstructed umbilicus to that of her native umbilicus before development of the lesion. However, because postoperative conformers have not been systematically investigated, the contribution of the conformer to the observed outcome cannot be distinguished from that of the surgical design, deep fascial fixation, natural scar maturation, or other postoperative factors. Future studies are needed to determine whether prolonged external support contributes to long-term maintenance of neoumbilical morphology.

Several limitations should be acknowledged. This is a single-patient case report, and the favorable outcome cannot establish superiority of this technique over previously described methods or demonstrate its general reproducibility. The proposed dimensions represent the surgical design used in this patient and should not be interpreted as fixed measurements applicable to all abdominal anatomies. Histopathological processing and slide preparation were performed by an external pathology laboratory; consequently, the original histopathology slides and photomicrographs were not available for independent review or inclusion in this manuscript, and the pathological findings were based on the official pathology report. Patient satisfaction was based on subjective postoperative assessment rather than a validated patient-reported outcome measure. Furthermore, the six-month follow-up period does not establish long-term durability of the reconstructed contour. Nevertheless, the favorable clinical outcome, combined with defined geometric planning, deep fascial fixation, and a detailed schematic description of the technique, may provide a practical framework for immediate neoumbilicoplasty following excision of selected benign umbilical lesions and warrants further evaluation in larger patient series with longer follow-up.

## Conclusions

Symptomatic umbilical lipomas requiring excision with loss or distortion of the native umbilical anatomy are uncommon, and evidence regarding immediate reconstruction following excision of benign umbilical tumors remains limited. In this case, en bloc excision followed by immediate neoumbilicoplasty using local tissue rearrangement and deep fascial fixation restored umbilical depth and contour and resulted in a favorable aesthetic outcome at six-month follow-up, with the patient reporting high satisfaction with the reconstructed umbilicus. The standardized geometric preoperative design, combined with intraoperative reassessment of skin redundancy after lesion excision and flap approximation, provided a structured yet adaptable approach to reconstruction. Deep fixation of the reconstructed umbilicus to the abdominal fascia was used to create a central depression and depth, while postoperative use of an umbilical conformer and abdominal compression provided additional external support during scar maturation. Although conclusions regarding the independent contribution of individual components of the technique cannot be drawn from a single case, this approach may provide a practical framework for immediate reconstruction following excision of selected superficial benign umbilical lesions. Larger studies with longer follow-up are needed to assess the reproducibility and long-term durability of the technique.
